# On the Analogy between Electrolytes and Ion-Generating Nanomaterials in Liquid Crystals

**DOI:** 10.3390/nano10030403

**Published:** 2020-02-25

**Authors:** Yuriy Garbovskiy

**Affiliations:** Department of Physics and Engineering Physics, Central Connecticut State University, New Britain, CT 06050, USA; ygarbovskiy@ccsu.edu or ygarbovskiy@gmail.com; Tel.: +1-860-832-2944

**Keywords:** liquid crystals, nanomaterials, ions, ion generation, analogy

## Abstract

Nanomaterials in liquid crystals are a hot topic of contemporary liquid crystal research. An understanding of the possible effects of nanodopants on the properties of liquid crystals is critical for the development of novel mesogenic materials with improved functionalities. This paper focuses on the electrical behavior of contaminated nanoparticles in liquid crystals. More specifically, an analogy between electrolytes and ion-generating nanomaterials in liquid crystals is established. The physical consequences of this analogy are analyzed. Under comparable conditions, the number of ions generated by nanomaterials in liquid crystals can be substantially greater than the number of ions generated by electrolytes of similar concentration.

## 1. Introduction

Ions in liquid crystals have been studied since the early 1960s because of their strong effects on the electrooptical response of mesogenic materials [1,2]. 

Early liquid-crystal display (LCD) technologies utilized the dynamic light scattering caused by electrohydrodynamic instabilities in nematic materials [3,4,5]. The presence of ions was essential for the effect [3,4,5]. As a result, ion-generating materials such as dissociating salts [6,7] were intentionally added to liquid crystals. The discovery of electrohydrodynamic instability in liquid crystals [3,4,5] enabled their early applications as light shutters [8,9], and simultaneously initiated very active research into the mechanisms of ion generation in liquid crystals [10,11,12,13,14]. The invention of modern thin-film transistor (TFT) LCD technologies placed an emphasis on the synthesis and electrical characterization of high-resistivity liquid crystals [15,16]. As the electric-field-induced orientational effect is at the heart of the TFT LCD operation, the presence of ions in liquid crystals is very undesirable. It can lead to many negative side effects, including image flickering, image sticking, and overall slow response [1,16]. Even though ions in liquid crystals became unwanted objects, research into the electrical properties of liquid crystals was very active because it enabled the selection of suitable mesogenic materials [17,18] and alignment layers [19,20,21,22]. It is worth mentioning that the effect of dynamic light scattering in liquid crystals was not totally abandoned. Recently, it found very promising applications in the development of dynamic shutters and smart windows [23,24,25,26,27,28]. 

An ongoing competition between LCD and alternative display technologies such as organic light-emitting diode displays [29] resulted in both (1) the improvement of existing LCD technologies and the development of advanced LCD technologies (liquid crystal on silicon (LCoS) displays for virtual and augmented reality [30]) and (2) a rapid growth of non-display applications of liquid crystals. The most well-known ones include photonic [31,32] and biophotonic applications [33], such as tunable lenses [34], filters for hyperspectral imaging [33], retarders [33], waveplates [35], and numerous LCoS devices [36,37,38].

Both display and non-display applications of liquid crystals rely on novel liquid-crystal materials with improved functionalities. Regardless of the type of liquid-crystal-based application, ions—typically present in liquid crystals in small quantities—can alter the performance of liquid crystal devices through the well-known screening effect [1,16]. Therefore, an understanding of possible sources of ion generation in liquid crystals is very important [39,40]. 

Recently, liquid crystals doped with nano-objects have emerged as novel tunable materials with advanced functionalities [41,42,43,44,45,46,47]. Electrical properties of liquid crystals doped with ferroelectric [48,49,50,51,52,53,54], magnetic [55,56,57], metal [58,59,60,61,62,63], semiconductor and dielectric [64,65,66,67,68,69], and carbon-based (fullerenes, carbon nanotubes, graphene, diamond) nanomaterials [70,71,72,73,74,75] were studied by many research teams (see also recent reviews [76,77] and references therein). The ion capturing effect is naturally expected for nanomaterials dispersed in liquid crystals. As a result, nanomaterials dispersed in liquid crystals can capture ions, thus providing a permanent purification of liquid crystals. At the same time, nanomaterials releasing ions in liquid crystals may seem like an unexpected possibility. Yet, many experiments have also confirmed the ion-generating properties of nanomaterials in liquid crystals [61,68,71,76,77]. The effect of ion generation by nanoparticles in liquid crystals can be very strong, as was reported recently by Urbanski and Lagerwall [61]. They found that the number of ions generated by functionalized gold nanoparticles dispersed in nematic liquid crystal 5CB can be comparable to and even greater than the number of ions generated in liquid crystals by 1:1 electrolytes [61]. The ionic contamination of ligands covering the surface of gold nanoparticles was considered a major reason for the observed effect [61]. The effects caused by the ionic contamination of nanomaterials were also modeled and applied to existing experimental data in a series of publications [78,79,80,81]. However, a comparison between the behavior of electrolytes and ion-generating nanoparticles in liquid crystals was not performed in papers [78,79,80,81]. Given the high promise of ion-generating nanomaterials for the development of liquid-crystal-based smart windows, it is important to consider and analyze the analogy between electrolytes and ion-generating nanomaterials in liquid crystals. The analysis of this analogy is the major objective of the present paper.

## 2. Model (Analogy between Ion-Generating Nanomaterials and Electrolytes in Liquid Crystals) 

To focus on ion-generating processes only, let us assume that liquid crystals are free of ions prior to mixing them with contaminated nanoparticles. Consider contaminated nanoparticles of a spherical shape dispersed in a liquid crystal host. Once contaminated nanomaterials are dispersed in liquid crystals, some ions will be released from the surface, thus enriching the bulk concentration of mobile ions *n* (for simplicity, symmetrical positive and negative ions of the volume concentration $n^{+}=n^{-}=n$ are assumed). At the same time, some of the released ions can also be recaptured by nanoparticles. The following rate Equation (1) can describe the aforementioned processes of ion generation (the second term of the equation, $k_{d}n_{NP}A_{NP}\sigma_{NP}\theta_{NP}$) and ion capturing processes (the first term of the equation, $k_{a}nn_{NP}A_{NP}\sigma_{NP}\left(1-\theta_{NP}\right)$),

(1) $$\frac{dn}{dt}=-k_{a}nn_{NP}A_{NP}\sigma_{NP}\left(1-\theta_{NP}\right)+k_{d}n_{NP}A_{NP}\sigma_{NP}\theta_{NP}$$

where $n_{NP}$ is the volume concentration of nanoparticles, $A_{NP}$ is their surface area, $\sigma_{NP}$ is the surface density of sites available for the ionic contaminants, $\theta_{NP}$ is the fractional surface coverage of contaminated nanoparticles, $k_{a}$ is a constant describing the ion capturing process (in the simplest case of a physical adsorption, this is an adsorption rate constant), and $k_{d}$ is the constant characterizing the ion generation process (in the simplest case of a physical adsorption, this is a desorption rate constant). 

Equation (1) should be solved together with Equation (2) representing the conservation of the total number of ions:

(2) $$n_{NP}A_{NP}\sigma_{NP}\nu_{NP}=n+n_{NP}A_{NP}\sigma_{NP}\theta_{NP}$$

where $\nu_{NP}$ is the dimensionless contamination factor of nanoparticles accounting for their ionic contamination [78]. By denoting $g=A_{NP}\sigma_{NP}\nu_{NP}$, substituting Equation (2) into Equation (1), and assuming $n_{NP}g\left(\frac{1}{\nu_{NP}}-1\right)\gg n$, one can get Equation (3):

(3) $$\frac{dn}{dt}=-\left[k_{d}+k_{a}n_{NP}g\left(\frac{1}{\nu_{NP}}-1\right)\right]n+k_{d}n_{NP}g$$

By applying initial conditions ($n=0\;m^{-3}$ t=0 s), its solution can be written as Equation (4):

(4) $$n=\frac{k_{d}n_{NP}g}{k_{d}+k_{a}n_{NP}g\left(\frac{1}{\nu_{NP}}-1\right)}\left[1-e^{-\left(k_{d}+k_{a}n_{NP}g\left(\frac{1}{\nu_{NP}}-1\right)\right)t}\right]$$

In the case of 1:1 symmetrical electrolytes in liquid crystals, the ion generation/ion recombination processes obey the well-known Equations (5)–(6) [82,83]:

(5) $$\frac{dn}{dt}=-k_{R}n^{2}+k_{D}\left(C_{0}-n\right)$$

(6) $$C_{0}=C+n$$

where $n^{+}=n^{-}=n$ is the volume concentration of mobile ions, C is the volume concentration of a non-dissociated salt and $C_{0}$ is its initial concentration, $k_{R}$ is the recombination rate constant, and $k_{D}$ is the dissociation rate constant.

Assuming $C_{0}\gg C$, Equations (5)–(6) can be rewritten as Equation (7):

(7) $$\frac{dn}{dt}=-\left(k_{R}C_{0}+k_{D}\right)n+k_{D}C_{0}$$

Applying initial conditions ($n=0\;m^{-3}$ if t=0 s), the solution of Equations (5)–(6) can be written as Equation (8):

(8) $$n=\frac{k_{D}C_{0}}{k_{D}+k_{R}C_{0}}\left[1-e^{-\left(k_{D}+k_{R}C_{0}\right)t}\right]$$

A striking similarity between Equation (4) and Equation (8) reveals an analogy between ion-generating nanoparticles and electrolytes in liquid crystals. This analogy is summarized in Table 1. 

According to Table 1, the desorption rate constant $k_{d}$ is analogous to the dissociation rate constant $k_{D}$; the total number of ions carried by ion-generating nanoparticles $n_{NP}g$ (where $g=A_{NP}\sigma_{NP}\nu_{NP}$) is equivalent to the initial concentration of electrolytes $C_{0}$; and the product $k_{a}\left(\frac{1}{\nu_{NP}}-1\right)$ is similar to the recombination rate constant $k_{R}$. As expected, fully contaminated nanomaterials ($\nu_{NP}=1$) are the most efficient ion-generating objects because of the zero-recombination coefficient ($k_{a}\left(\frac{1}{\nu_{NP}}-1\right)=0$). At the same time, 100% pure nanomaterials ($\nu_{NP}=0$) are characterized by an effectively infinite recombination coefficient ($k_{a}\left(\frac{1}{\nu_{NP}}-1\right)\overset{\upsilon_{NP}=0}{\to }\infty$). As a result, they cannot generate ions and act as ion-trapping objects.

## 3. Results and Discussion

In the case of electrolytes in liquid crystals, the molar concentration $c_{el}$ (mol/L) is typically used. The weight concentration $\omega_{NP}$ is a convenient measure of the amount of nanomaterials dispersed in liquid crystals. Equations (1)–(8) are written assuming the volume concentration *n* ($n_{NP}$ or *C*) (m^-3^). In the limit of relatively low concentrations, the volume concentration of nanomaterials is related to their weight concentration via equation nNP≈ωNP(ρNPρLCVNP), and the molar concentration (mol/L) of nanomaterials can be written as cNP=10−3(nNPNA), where $\rho_{NP}$ is the volumetric mass density of nanoparticles, $\rho_{LC}$ is the volumetric mass density of liquid crystals, $V_{NP}$ is the volume of a single nanoparticle, and $N_{A}$ is the Avogadro constant. In the case of a spherical nanoparticle, its volume is related to its radius as $V_{NP}=\frac{4}{3}\pi R_{NP}^{3}$.

**Figure 1:**
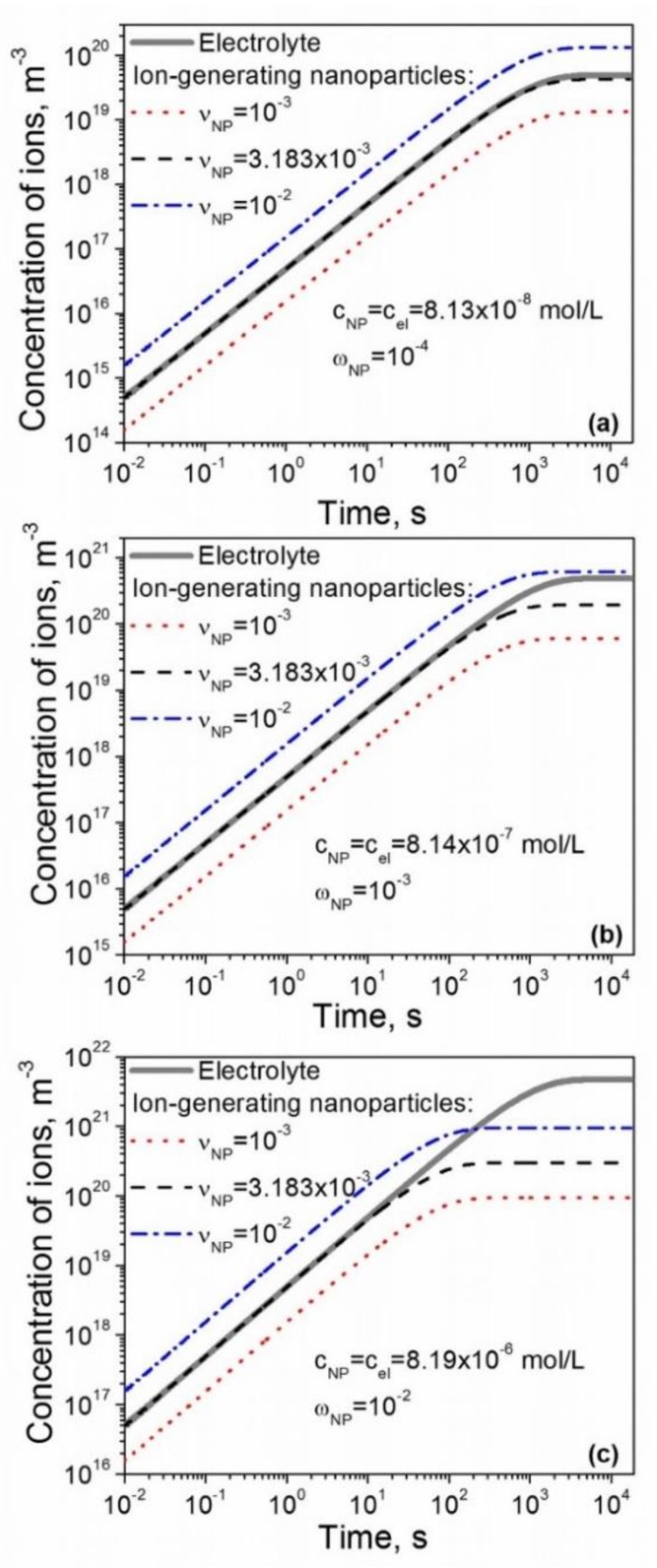
The concentration of ions generated in liquid crystals by electrolytes (solid curves) and contaminated nanomaterials (dotted, dashed, and dash-dotted curves) as a function of time. The concentrations of nanomaterials and electrolytes are: (**a**) $c_{NP}=c_{el}=8.13\times 10^{-8}\;\mathrm{mol}/L$; (**b**) $c_{NP}=c_{el}=8.14\times 10^{-7}\;\mathrm{mol}/L$; (**c**) $c_{NP}=c_{el}=8.19\times 10^{-6}\;\mathrm{mol}/L$. Physical parameters: *R_NP_* = 5 nm; $\sigma_{NP}$ = 10^18^ m^-2^; *k_a_* = *k_R_* = 10^-26^ m^3^/s; *k_d_* = *k_D_* = 10^-3^ s^-1^; $\frac{\rho_{NP}}{\rho_{LC}}$ = 3.9.

The ion-generating properties of electrolytes and contaminated nanomaterials in liquid crystals can be reasonably compared if they are characterized by the same molar concentration $c_{el}=c_{NP}$ and similar rate constants $k_{d}=k_{D}$ and $k_{a}=k_{R}$. The time dependence of the concentration of ions generated in liquid crystals by both contaminated nanomaterials and electrolytes of the same molar concentration is shown in Figure 1. The concentration of ions was computed for several concentrations of dopants normally used in experiments ($c_{NP}=c_{el}=8.13\times 10^{-8}\;\mathrm{mol}/L$ (Figure 1a), $c_{NP}=c_{el}=8.14\times 10^{-7}\;\mathrm{mol}/L$ (Figure 1b), and $c_{NP}=c_{el}=8.19\times 10^{-6}\;\mathrm{mol}/L$ (Figure 1c). To account for a reasonable level of ionic contamination of nanomaterials, the contamination factors were chosen to be $\nu_{NP}=10^{-2}$ (dash-dotted curve), $\nu_{NP}=10^{-3}$ (dotted curve), and $\nu_{NP}=3.183\times 10^{-3}$ (dashed curve) (Figure 1).

In all cases shown in Figure 1, depending on the level of the ionic contamination $\nu_{NP}$, the number of generated ions by nanomaterials in liquid crystals could be smaller than (dotted curves), comparable to (dashed curves), or even greater than (dash-dotted curves) the number of ions generated by electrolytes (solid curves). The time dependences in Figure 1 are characterized by time constants. For the period of time longer than the time constant, the concentration of ions in liquid crystals reaches a steady-state value (Figure 1). In the case of liquid crystals doped with nanomaterials (Figure 1, dashed, dotted, and dash-dotted curves), this time constant $\tau_{NP}$ can be written as Equation (9):

(9) $$\tau_{NP}=\frac{1}{k_{d}+k_{a}n_{NP}g\left(\frac{1}{\nu_{NP}}-1\right)}$$

The time constant $\tau_{el}$ of liquid crystals doped with electrolytes (Figure 1, solid curve) is expressed by Equation (10):

(10) $$\tau_{el}=\frac{1}{k_{D}+k_{R}C_{0}}$$

**Figure 2:**
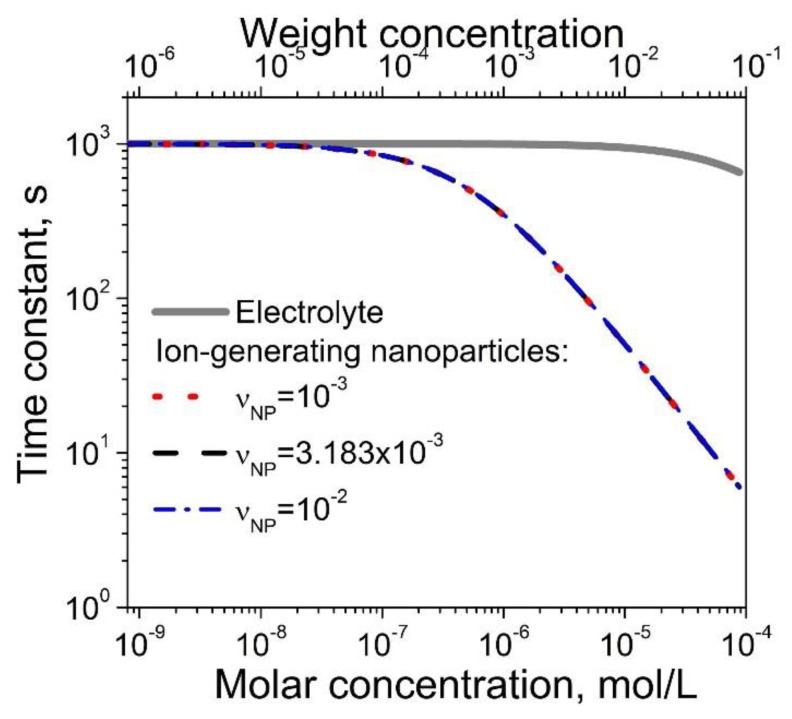
Time constants $\tau_{el}$ (solid curve) and $\tau_{NP}$ (dotted, dashed, and dash-doted curves) versus the concentration of ion-generating materials (either electrolytes or contaminated nanoparticles). Physical parameters: *R_NP_* = 5 nm; $\sigma_{NP}$ = 10^18^ m^-2^; *k_a_* = *k_R_* = 10^-26^ m^3^/s; *k_d_* = *k_D_* = 10^-3^ s^-1^; $\frac{\rho_{NP}}{\rho_{LC}}$ = 3.9.

For given materials, the time constants $\tau_{NP}$ and $\tau_{el}$ can be controlled by changing the concentration of nanomaterials and electrolytes. Higher concentrations of ion-generating materials correspond to smaller values of time constants. Interestingly, under comparable conditions, the time constant $\tau_{NP}$ as a function of the molar concentration exhibits a more rapid decrease compared to the same dependence $\tau_{el}\left(c_{el}\right)$ for electrolytes in liquid crystals (see Figure 2, where electrolytes in liquid crystals are represented by a solid curve). 

Figure 1 and Figure 2 indicate that both the transient and steady-state concentration of ions in liquid crystals can be controlled by changing the concentration of ion-generating nanomaterials or electrolytes. The use of nanomaterials offers one more level of control over the generated ions by changing the contamination factor $\nu_{NP}$ (Figure 1 and Figure 2). 

**Figure 3:**
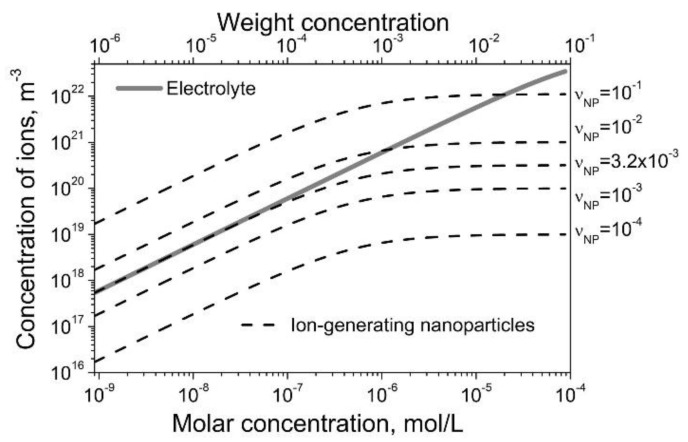
Steady-state concentration of ions generated in liquid crystals by electrolytes (solid curve) and contaminated nanomaterials (dashed curves) as a function of their molar/weight concentration. Physical parameters: *R_NP_* = 5 nm; $\sigma_{NP}$ = 10^18^ m^-2^; *k_a_* = *k_R_* = 10^-26^ m^3^/s; *k_d_* = *k_D_* = 10^-3^ s^-1^; $\frac{\rho_{NP}}{\rho_{LC}}$ = 3.9.

The comparison of electrolytes and ion-generating nanomaterials in the steady-state regime is shown in Figure 3. The concentration of generated ions in liquid crystals by contaminated nanomaterials can be controlled within a broad range. Interestingly, this concentration can even exceed the number of ions generated in liquid crystals by electrolytes, as shown by dashed curves in Figure 3 (for $\nu_{NP}\geq 3.2\times 10^{-3}$).

## 4. Conclusions

The ionic contamination of nanomaterials can result in very unusual effects. Once dispersed in liquid crystals, contaminated nanomaterials can act as ion-generating objects. Ion-generating nanomaterials represent a new source of ion generation in liquid crystals. Given the important role of both ions and nanomaterials for the development of advanced liquid crystal technology, the possibility of ion-generating behavior of nanomaterials in liquid crystals should not be ignored. 

In this paper, a simple analogy between electrolytes and ion-generating nanomaterials in liquid crystals was established (Equations (1)–(8) and Table 1). This analogy allowed for a quantitative prediction of the ion generation in liquid crystals by means of contaminated nanomaterials. In addition, it also revealed some advantages of using ion-generating nanomaterials for liquid crystal applications requiring the presence of ions. Under certain conditions, ion-generating nanomaterials can generate ions in liquid crystals more efficiently than typical electrolytes. More specifically, the steady-state concentration of ions generated in liquid crystals by nanomaterials can be reached faster (Figure 2), and it can be greater than the same quantity in the case of electrolytes in liquid crystals (Figure 3). 

Some limitations of the presented analogy should also be mentioned. The analogy between ion-generating nanomaterials and electrolytes in liquid crystals relies on rate Equation (1). As was already discussed in previous publications [84,85,86], this rate equation is valid in the regime of relatively low concentrations. Typically, such low concentrations are common for thermotropic liquid crystals, thus justifying the established analogy. In the case of high concentrations, a more rigorous model utilizing the Poisson–Boltzmann equation should be considered [87,88,89,90].

## Figures and Tables

**Table 1:** Analogy between ion-generating nanomaterials and electrolytes in liquid crystals.

Ion-Generating Nanomaterials	Electrolytes
*n*	*n*
$k_{d}$	$k_{D}$
$n_{NP}g$ (where $g=A_{NP}\sigma_{NP}\nu_{NP}$)	$C_{0}$
$k_{a}\left(\frac{1}{\nu_{NP}}-1\right)$	$k_{R}$

## References

[1] Neyts K., Beunis F. (2014). Handbook of Liquid Crystals: Physical Properties and Phase Behavior of Liquid Crystals.

[2] Colpaert C., Maximus B., Meyere D. (1996). Adequate measuring techniques for ions in liquid crystal layers. Liq. Cryst..

[3] Williams R. (1963). Domains in Liquid Crystals. J. Chem. Phys..

[4] Blinov L.M. (1986). Electrohydrodynamic effects in liquid crystals. Sci. Prog. (1933-).

[5] Kramer L., Pesch W., Buka A., Kramer L. (1996). Electrohydrodynamic instabilities in nematic liquid crystals. Pattern Formation in Liquid Crystals.

[6] Chang R., Richardson J.M. (1973). The anisotropic electrical conductivity of MBBA containing tetrabutyl-ammonium tetraphenyl-boride. Mol. Cryst. Liq. Cryst..

[7] Barnik M.I., Blinov L.M., Grebenkin M.F., Pikin S.A., Chigrinov V.G. (1976). Electrohydrodynamic instability in nematic liquid crystals. Sov. Phys. JETP.

[8] Heilmeier G.H., Zanoni L.A., Barton L.A. (1968). Dynamic scattering in nematic liquid crystals. Appl. Phys. Lett..

[9] Heilmeier G.H., Zanoni L.A., Barton L.A. (1968). Dynamic scattering: A new electrooptic effect in certain classes of nematic liquid crystals. Proc. IEEE.

[10] Chieu T.C., Yang K.H. (1989). Transport properties of ions in ferroelectric liquid crystal cells. Jpn. J. Appl. Phys..

[11] Kovalchuk A.V., Lavrentovich O.D., Linev V.A. (1988). Electrical conductivity of γ-irradiated cholesteric liquid crystals. Sov. Tech. Phys. Lett..

[12] Murakami S., Naito H. (1997). Charge injection and generation in nematic liquid crystal cells. Jpn. J. Appl. Phys..

[13] De Vleeschouwer H., Verschueren A., Bougrioua F., Van Asselt R., Alexander E., Vermael S., Neyts K., Pauwels H. (2001). Long-term ion transport in nematic liquid crystal displays. Jpn. J. Appl. Phys..

[14] Murakami S., Naito H. (1997). Electrode and interface polarizations in nematic liquid crystal cells. Jpn. J. Appl. Phys..

[15] Koide N. (2014). The Liquid Crystal Display Story. 50 Years of Liquid Crystal r&d that Lead the Way to the Future.

[16] Chigrinov V.G. (1999). Liquid Crystal Devices: Physics and Applications.

[17] Naemura S., Sawada A. (2003). Ionic conduction in nematic and smectic liquid crystals. Mol. Cryst. Liq. Cryst..

[18] Hung H.Y., Lu C.W., Lee C.Y., Hsu C.S., Hsieh Y.Z. (2012). Analysis of metal ion impurities in liquid crystals using high resolution inductively coupled plasma mass spectrometry. Anal. Methods.

[19] Mizusaki M., Enomoto S., Hara Y. (2017). Generation mechanism of residual direct current voltage for liquid crystal cells with polymer layers produced from monomers. Liq. Cryst..

[20] Huang Y., Bhowmik A., Bos P.J. (2012). The effect of salt on ion adsorption on a SiO_x_ alignment film and reduced conductivity of a liquid crystal host. J. Appl. Phys..

[21] Kobayashi S., Xu J., Furuta H., Murakami Y., Kawamoto S., Ohkouchi M., Hasebe H., Takatsu H. (2004). Fabrication and electro-optic characteristics of polymer-stabilized V-mode ferroelectric liquid crystal display and intrinsic H-V-mode ferroelectric liquid crystal displays: Their application to field sequential full colour active matrix liquid crystal displays. Opt. Eng..

[22] Huang Y., Bhowmik A., Bos P.J. (2012). Characterization of ionic impurities adsorbed onto a 5° SiO_x_ alignment film. Jpn. J. Appl. Phys..

[23] Geis M.W., Bos P.J., Liberman V., Rothschild M. (2016). Broadband optical switch based on liquid crystal dynamic scattering. Opt. Express.

[24] Dabrowski R., Dziaduszek J., Bozetka J., Piecek W., Mazur R., Chrunik M., Perkowski P., Mrukiewicz M., Żurowska M., Weglowska D. (2017). Fluorinated smectics–New liquid crystalline medium for smart windows and memory displays. J. Mol. Liq..

[25] Konshina E.A., Shcherbinin D.P. (2018). Study of dynamic light scattering in nematic liquid crystal and its optical, electrical and switching characteristics. Liq. Cryst..

[26] Madhuri P.L., Martin-Palma R.J., Martín-Adrados B., Abdulhalim I. (2019). Voltage controlled scattering from porous silicon Mie-particles in liquid crystals. J. Mol. Liq..

[27] Abdulhalim I., Madhuri P., Diab M., Mokari T. (2019). Novel easy to fabricate liquid crystal composite with potential for electrically or thermally controlled transparency windows. Opt. Express.

[28] Zhan Y., Lu H., Jin M., Zhou G. (2019). Electrohydrodynamic instabilities for smart window applications. Liq. Cryst..

[29] Chen H.W., Lee J.H., Lin B.Y., Chen S., Wu S.T. (2018). Liquid crystal display and organic light-emitting diode display: Present status and future perspectives. Light Sci. Appl..

[30] Huang Y., Liao E., Chen R., Wu S.-T. (2018). Liquid-Crystal-on-Silicon for Augmented Reality Displays. Appl. Sci..

[31] Chigrinov V.G. (2014). Liquid Crystal Photonic.

[32] He Z., Tan G., Chanda D., Wu S.-T. (2019). Novel liquid crystal photonic devices enabled by two-photon polymerization [Invited]. Opt. Express.

[33] Abdulhalim I. (2011). Non-display bio-optic applications of liquid crystals. Liq. Cryst. Today.

[34] Lin Y., Wang Y., Reshetnyak V. (2017). Liquid crystal lenses with tunable focal length. Liq. Cryst. Rev..

[35] De Sio L., Roberts D.E., Liao Z., Hwang J., Tabiryan N., Steeves D.M., Kimball B.R. (2018). Beamshaping diffractive wave plates. Appl. Opt..

[36] Zhang Z., You Z., Chu D. (2014). Fundamentals of phase-only liquid crystal on silicon (LCOS) devices. Light Sci. Appl..

[37] Lazarev G., Chen P.-J., Strauss J., Fontaine N., Forbes A. (2019). Beyond the display: Phase-only liquid crystal on Silicon devices and their applications in photonics. Opt. Express.

[38] Otón J.M., Otón E., Quintana X., Geday M.A. (2018). Liquid-crystal phase-only devices. J. Mol. Liq..

[39] Garbovskiy Y. (2018). Time-dependent electrical properties of liquid crystal cells: Unravelling the origin of ion generation. Liq. Cryst..

[40] Korniychuk P.P., Gabovich A.M., Singer K., Voitenko A.I., Reznikov Y.A. (2010). Transient and steady electric currents through a liquid crystal cell. Liq. Cryst..

[41] Stamatoiu O., Mirzaei J., Feng X., Hegmann T. (2012). Nanoparticles in liquid crystals and liquid crystalline nanoparticles. Top. Curr. Chem..

[42] Garbovskiy Y., Glushchenko A. (2010). Liquid crystalline colloids of nanoparticles: Preparation, properties, and applications. Solid State Phys..

[43] Bisoyi H.K., Kumar S. (2011). Liquid-crystal nanoscience: An emerging avenue of soft self-assembly. Chem. Soc. Rev..

[44] Lagerwall J.P.F., Scalia G. (2016). Liquid Crystals with Nano and Microparticles.

[45] Shen Y., Dierking I. (2019). Perspectives in Liquid-Crystal-Aided Nanotechnology and Nanoscience. Appl. Sci..

[46] Dierking I. (2018). Nanomaterials in Liquid Crystals. Nanomaterials.

[47] Dierking I. (2019). From colloids in liquid crystals to colloidal liquid crystals. Liq. Cryst..

[48] Shukla R.K., Liebig C.M., Evans D.R., Haase W. (2014). Electro-optical behaviour and dielectric dynamics of harvested ferroelectric LiNbO3 nanoparticle-doped ferroelectric liquid crystal nanocolloids. RSC Adv..

[49] Basu R., Garvey A. (2014). Effects of ferroelectric nanoparticles on ion transport in a liquid crystal. Appl. Phys. Lett..

[50] Garbovskiy Y., Glushchenko I. (2015). Ion trapping by means of ferroelectric nanoparticles, and the quantification of this process in liquid crystals. Appl. Phys. Lett..

[51] Hsiao Y.C., Huang S.M., Yeh E.R., Lee W. (2016). Temperature dependent electrical and dielectric properties of nematic liquid crystals doped with ferroelectric particles. Displays.

[52] Kumar P., Debnath S., Rao N.V., Sinha A. (2018). Nanodoping: A route for enhancing electro-optic performance of bent core nematic system. J. Phys. Condens. Matter.

[53] Al-Zangana S., Turner M., Dierking I. (2017). A comparison between size dependent paraelectric and ferroelectric BaTiO_3_ nanoparticle doped nematic and ferroelectric liquid crystals. J. Appl. Phys..

[54] Garbovskiy Y., Glushchenko A. (2017). Ferroelectric Nanoparticles in Liquid Crystals: Recent Progress and Current Challenges. Nanomaterials.

[55] Sharma K.P., Malik P., Raina K.K. (2016). Electro-optic, dielectric and optical studies of NiFe_2_O_4_-ferroelectric liquid crystal: A soft magnetoelectric material. Liq. Cryst..

[56] Mertelj A., Lisjak D. (2017). Ferromagnetic nematic liquid crystals. Liq. Cryst. Rev..

[57] Pandey F.P., Rastogi A., Manohar R., Dhar R., Singh S. (2019). Dielectric and electro-optical properties of zinc ferrite nanoparticles dispersed nematic liquid crystal 4’-Heptyl-4-biphenylcarbonnitrile. Liq. Cryst..

[58] Urbanski M., Lagerwall J. (2016). Nanoparticles dispersed in liquid crystals: Impact on conductivity, low-frequency relaxation and electro-optical performance. J. Mater. Chem. C.

[59] Middha M., Kumar R., Raina K.K. (2016). Photoluminescence tuning and electro-optical memory in chiral nematic liquid crystals doped with silver nanoparticles. Liq. Cryst..

[60] Podgornov F.V., Wipf R., Stuhn B., Ryzhkova A.V., Haase W. (2016). Low-frequency relaxation modes in ferroelectric liquid crystal/gold nanoparticle dispersion: Impact of nanoparticle shape. Liq. Cryst..

[61] Urbanski M., Lagerwall J.P.F. (2017). Why organically functionalized nanoparticles increase the electrical conductivity of nematic liquid crystal dispersions. J. Mater. Chem. C.

[62] Podgornov F.V., Gavrilyak M., Karaawi A., Boronin V., Haase W. (2018). Mechanism of electrooptic switching time enhancement in ferroelectric liquid crystal/gold nanoparticles dispersion. Liq. Cryst..

[63] Shivaraja S.J., Gupta R.K., Kumar S., Manjuladevi V. (2019). Effect of functionalised silver nanoparticle on the elastic constants and ionic transport of a nematic liquid crystal. Liq. Cryst..

[64] Chandran A., Prakash J., Gangwar J., Joshi T., Srivastava A.K., Haranath D., Biradar A.M. (2016). Low-voltage electro-optical memory device based on NiO nanorods dispersed in a ferroelectric liquid crystal. RSC Adv..

[65] Ha Y.-S., Kim H., Park H.-G., Seo D.S. (2012). Enhancement of electrooptic properties in liquid crystal devices via titanium nanoparticle doping. Opt. Express.

[66] Shcherbinin D.P., Konshina E.A. (2017). Ionic impurities in nematic liquid crystal doped with quantum dots CdSe/ZnS. Liq. Cryst..

[67] Konshina E., Shcherbinin D., Kurochkina M. (2018). Comparison of the properties of nematic liquid crystals doped with TiO_2_ and CdSe/ZnS nanoparticles. J. Mol. Liq..

[68] Shcherbinin D.P., Konshina E.A. (2017). Impact of titanium dioxide nanoparticles on purification and contamination of nematic liquid crystals. Beilstein J. Nanotechnol..

[69] Prakasha J., Khana S., Chauhana S., Biradar A.M. (2019). Metal oxide-nanoparticles and liquid crystal composites: A review of recent progress. J. Mol. Liq..

[70] Lee C.W., Shih W.P. (2010). Quantification of ion trapping effect of carbon nanomaterials in liquid crystals. Mater. Lett..

[71] Tomylko S., Yaroshchuk O., Kovalchuk O., Maschke U., Yamaguchi R. (2012). Dielectric properties of nematic liquid crystal modified with diamond nanoparticles. Ukr. J. Phys..

[72] Samoilov A.N., Minenko S.S., Fedoryako A.P., Lisetski L.N., Lebovka N.I., Soskin M.S. (2014). Multiwalled vs. single-walled carbon nanotube dispersions in nematic liquid crystals: Comparative studies of optical transmission and dielectric properties. Funct. Mater..

[73] Jian B.R., Tang C.Y., Lee W. (2011). Temperature-dependent electrical properties of dilute suspensions of carbon nanotubes in nematic liquid crystals. Carbon.

[74] Wu P.C., Lisetski L.N., Lee W. (2015). Suppressed ionic effect and low-frequency texture transitions in a cholesteric liquid crystal doped with graphene nanoplatelets. Opt. Express.

[75] Yadav S.P., Singh S. (2016). Carbon nanotube dispersion in nematic liquid crystals: An overview. Prog. Mater. Sci..

[76] Garbovskiy Y., Glushchenko I. (2015). Nano-objects and ions in liquid crystals: Ion trapping effect and related phenomena. Crystals.

[77] Garbovskiy Y. (2018). Nanomaterials in Liquid Crystals as Ion-Generating and Ion-Capturing Objects. Crystals.

[78] Garbovskiy Y. (2016). Switching between purification and contamination regimes governed by the ionic purity of nanoparticles dispersed in liquid crystals. Appl. Phys. Lett..

[79] Garbovskiy Y. (2016). Electrical properties of liquid crystal nano-colloids analysed from perspectives of the ionic purity of nano-dopants. Liq. Cryst..

[80] Garbovskiy Y. (2017). Nanoparticle enabled thermal control of ions in liquid crystals. Liq. Cryst..

[81] Garbovskiy Y. (2018). Nanoparticle—Enabled Ion Trapping and Ion Generation in Liquid Crystals. Adv. Condens. Matter Phys..

[82] Briere G., Gaspard F., Herino R. (1971). Ionic residual conduction in the isotropic phase of a nematic liquid crystal. Chem. Phys. Lett..

[83] Blinov L.M. (2010). Structure and Properties of Liquid Crystals.

[84] Garbovskiy Y. (2016). Adsorption/desorption of ions in liquid crystal nano-colloids: The applicability of the Langmuir isotherm, impact of high electric fields, and effects of the nanoparticle’s size. Liq. Cryst..

[85] Garbovskiy Y. (2017). Ions and size effects in nanoparticle/liquid crystal colloids sandwiched between two substrates. The case of two types of fully ionized species. Chem. Phys. Lett..

[86] Garbovskiy Y. (2018). Kinetics of Ion-Capturing/Ion-Releasing Processes in Liquid Crystal Devices Utilizing Contaminated Nanoparticles and Alignment Films. Nanomaterials.

[87] Barbero G., Evangelista L.R. (2006). Adsorption Phenomena and Anchoring Energy in Nematic Liquid Crystals.

[88] Steffen V., Cardozo-Filho L., Silva E.A., Evangelista L.R., Guirardello R., Mafra M.R. (2015). Equilibrium modeling of ion adsorption based on Poisson–Boltzmann equation. Colloids Surf. A.

[89] Batalioto F., Figueiredo Neto A.M., Barbero G. (2017). Ion trapping on silica nanoparticles: Effect on the ζ-potential. J. App. Phys..

[90] Steffen V., Silva E.A., Evangelista L.R., Cardozo-Filho L. (2018). Debya-Huckel approximation for simplification of ions adsorption equilibrium model based on Poisson-Boltzmann equation. Surf. Interfaces.

